# Ferroptosis-Related Hub Genes in Hepatocellular Carcinoma: Prognostic Signature, Immune-Related, and Drug Resistance Analysis

**DOI:** 10.3389/fgene.2022.907331

**Published:** 2022-07-22

**Authors:** Wei Wang, Fan Pan, Xinrong Lin, Jiakai Yuan, Chunyu Tao, Rui Wang

**Affiliations:** ^1^ Department of Medical Oncology, Jinling Hospital, Nanjing Medical University, Nanjing, China; ^2^ Department of Medical Oncology, School of Medicine, Jinling Hospital, Nanjing University, Nanjing, China

**Keywords:** ferroptosis, hepatocellular carcinoma, prognostic model, immune, drug resistance, bioinformatics analysis

## Abstract

**Background:** Hepatocellular carcinoma (HCC) is the most prevalent type of primary liver cancer with a high fatality rate and dismal prognosis because of frequent recurrence and lack of efficient therapies. Ferroptosis is a recently recognized iron-dependent cell death distinct from necroptosis and apoptosis. The relationship between ferroptosis-related hub gene expression and prognosis in HCC remains to be further elucidated.

**Methods:** Ferroptosis-related genes from the FerrDb database and the mRNA sequencing data and clinical information of HCC patients were obtained from The Cancer Genome Atlas (TCGA) database. The least absolute shrinkage and selection operator (LASSO) Cox regression was applied to identify a prognostic signature consisting of five ferroptosis-related hub genes in the TCGA cohort. The International Cancer Genome Consortium (ICGC) database was utilized to validate the reliability of the signature. Functional enrichment and immune-related analysis, including single-sample gene set enrichment analysis (ssGSEA), immune checkpoints, TIP-related genes, tumor stemness, and m6A-related genes, were performed to analyze the underlying mechanism. Additionally, the correlations between ferroptosis and drug resistance were evaluated using the NCI-60 database.

**Results:** A 5–hub-gene signature associated with ferroptosis was constructed by multivariate Cox regression analysis to stratify patients into two risk groups. Patients with high risk had worse prognosis than those with low risk. Multivariate Cox regression analysis uncovered that the risk score was an independent prognostic indicator. We also proved the signature’s predictive capacity using the Kaplan–Meier method and receiver operating characteristic (ROC) curve analysis. Functional analysis showed that nuclear division and the cell cycle were enriched. Immune-related analysis revealed that the signature was enriched in immune-related pathways. Moreover, the risk signature was significantly associated with immune cell infiltration, immune checkpoints, TIP-related genes, tumor stem cells, as well as m6A-related genes. Furthermore, these genes were crucial regulators of drug resistance.

**Conclusion:** We identified and validated a novel hub gene signature that is closely associated with ferroptosis as a new and efficient biomarker with favorable potential for predicting the prognosis of HCC patients. In addition, it also offers new insights into the molecular mechanisms of HCC and provides an effective approach for the treatment of HCC. Further studies are necessary to validate the results of our study.

## Introduction

Hepatocellular carcinoma (HCC) is a major type of adult liver malignancy. The latest global cancer statistics suggest that primary liver cancer is the sixth most commonly diagnosed cancer and the third leading cause of cancer death, with about 906,000 new cases and 830,000 deaths (8.3%) in 2020 (Sung et al., 2021). The major risk factors include hepatitis virus (hepatitis B virus, HBV, or hepatitis C virus, HCV) infection, heavy alcohol intake, nonalcoholic fatty liver disease, type 2 diabetes, and dietary toxins (aflatoxins and aristolochic acid) (Yang et al., 2019). Moreover, HCC shows high heterogeneity. As a result, many molecular targeted anticancer therapies are ineffective or even face resistance from some patients (Cancer Genome Atlas Research Network, 2017). The prognosis of HCC is very poor, with a 5-year survival rate of 14.1% (Allemani et al., 2018). At present, the diagnosis and treatment of HCC are not satisfactory.

In general, 80% of advanced HCC patients miss opportunities for surgery and ablation, but palliative treatments for HCC exhibit limited efficacy (Liu and Qin, 2019). In recent years, increasing attention has been paid to immunotherapy, and animal experiments and clinical trials have confirmed that immunotherapy plays an imperative role in the treatment of HCC patients (Zongyi and Xiaowu, 2020). Immunotherapy has been proven to be safe and effective for HCC treatment, which includes vaccines, immune checkpoint blockade, and adoptive cell transfer (ACT) (Li et al., 2015). The advent of immunotherapy has shed new light on the therapeutic strategies of HCC. Currently, immune checkpoint inhibitor (ICI) monotherapy using drugs such as nivolumab, pembrolizumab, and camrelizumab are primarily used for the second-line treatment of HCC patients at an advanced stage, and a series of relevant clinical trials are being conducted (El-Khoueiry et al., 2017; Llovet et al., 2018; Qin et al., 2020). Therefore, there is an urgent need to explore immunity-related analysis which can lay the foundation stone for immunotherapy treatment of HCC.

Ferroptosis is a newly discovered type of programmed cell death modality which is iron-dependent and mediated by lipid peroxidation. Typical morphological features include membrane rupture and blebbing, normal-sized nuclei without chromatin condensation, and mitochondrial changes, such as size reduction, increased membrane density, decreased or disappearance of mitochondrial cristae, and disruption of the mitochondrial outer membranes (Dixon et al., 2012). Extensive evidence has demonstrated that ferroptosis is closely associated with many diseases, such as cancer (Liang et al., 2019; Perez et al., 2020). It also plays a very critical role in gastric cancer, colorectal cancer, pancreatic cancer, and especially HCC (Nie et al., 2018). It has been reported that ferroptosis has become a promising treatment option for cancer cell death, especially for HCC resistant to traditional treatment (Tang et al., 2020; Yu and Wang, 2021). For instance, sorafenib could hinder cystine–glutamate antiporter and result in glutathione depletion, which induces ferroptosis in HCC cells (Hassannia et al., 2019; Liang et al., 2019). Emerging evidence suggests that ferroptosis can improve the immunotherapy response and inhibit tumor progression. Wang and coauthors have found that immunotherapy-activated CD8^+^ T cells could enhance ferroptosis by downregulating the expression of SLC7A11 and SLC3A2. The activation of ferroptosis further contributes to the anti-tumor effect of immunotherapy (Wang et al., 2019). However, a comprehensive analysis of the relationship between ferroptosis and immunotherapy response in HCC is not well characterized.

In this study, we obtained RNA expression data and clinical information from the TCGA and ICGC databases and analyzed them using bioinformatics tools. Thereafter, we constructed a protein–protein interaction (PPI) network to screen for hub genes. Next, we constructed a ferroptosis-related hub gene signature in the TCGA cohort, and the ICGC cohort was used to verify the reliability of the prognostic signature. In addition, functional enrichment analysis was conducted based on differentially expressed ferroptosis–related genes (FRGs) between the high-risk and low-risk groups. Finally, we further performed immune, tumor stemness, N6-methyladenosine (m6A) mRNA status, drug sensitivity, and immunohistochemical analysis. All of these might not only offer insight into the molecular mechanisms that participate in the tumorigenesis and progression of HCC but also provide an efficient method to predict the outcomes in HCC patients as well as contribute to selecting effective immunotherapy for HCC patients based on biomarkers.

## Materials and Methods

### Data Collection

The mRNA expression data [level 3; fragment per kilobase million (FPKM) normalized] from 374 tumor samples and 50 adjacent normal samples with corresponding clinicopathological information was downloaded from The Cancer Genome Atlas (TCGA) database (https://portal.gdc.cancer.gov/). RNA-seq data and clinical information of another cohort with 260 patients were obtained from the International Cancer Genome Consortium (ICGC) database (https://dcc.icgc.org/). After removing patients without significant clinical information, a total of 371 HCC patients in the TCGA database were included in the training cohort, and 260 patients in the ICGC database were included in the validation cohort. At the same time, the 382 ferroptosis-related genes were downloaded from the FerrDb database (http://www.zhounan.org/ferrdb/). All data analyzed in this study were publicly accessible. Ethics committee approval was not required.

### Model Establishment and Validation of Prognostic Ferroptosis-Related Hub Gene Signatures

The differentially expressed genes (DEGs) related to ferroptosis between tumor tissues and adjacent normal tissues were screened out by the “limma” package (Ritchie et al., 2015) using the Wilcoxon test in the TCGA cohort. The cutoff values were determined according to the parameters, *p* < 0.05 and false discovery rate (FDR) < 0.05. A univariate Cox analysis of overall survival (OS) was conducted to determine prognostic FRGs. The intersection of ferroptosis-related DEGs and prognostic genes was demonstrated using the “venn” R package.

An interaction network for the prognostic ferroptosis-related DEGs was plotted by the STRING (Search Tool for the Retrieval of Interacting Genes) database (version 11.0) (Szklarczyk et al., 2019). Then, we applied Cytoscape MCODE (Molecular Complex Detection) for screening hub genes (Jin et al., 2015). In addition, the MCODE app in Cytoscape software (version 3.9.1) was applied to check modules of the PPI network (degree cutoff = 2, max. depth = 100, k-core = 2, and node score cutoff = 0.2). The top ranked 10 genes in all modules were considered to be the hub genes. The expressions of these genes in tumor and normal samples were visualized using the “pheatmap” package in R.

The R packages “glmnet” and “survival” were utilized to further develop a prognostic risk signature with the least absolute shrinkage and selection operator (LASSO) method (Tibshirani, 1997; Simon et al., 2011).

The risk score was calculated based on the normalized expression level of each gene and its corresponding regression coefficients. The formula was as follows: risk score = $\overset{j}{\underset{n=1}{\sum }}(Coefj*Xj$ ), with *Coefj* representing the coefficient and *Xj* representing the expression level of each selected gene. We stratified patients into low- and high-risk subgroups according to the risk score. To detect internal correlation in these two groups, principal component analysis (PCA) and t-distributed stochastic neighbor embedding (t-SNE) were carried out using R packages “stats” and “Rtsne.” Survival analysis between the two subgroups was conducted through R package “surviminer” using the Kaplan–Meier curve and log-rank test. The predictive power of the gene signature was verified by the receiver operator characteristics (ROC) curve using the R package “timeROC.”

### Nomogram Construction and Assessment

Based on the variables identified with the univariate and multivariate Cox regression analyses, we also constructed the nomogram based on our prognostic gene signature using the R package “rms.” The calibration curve was plotted to assess the fitting and predictive ability of our prognostic model.

### Functional Enrichment Analysis

The DEGs between the low- and high-risk groups were screened out using the criteria: |log2FC| ≥ 1 and FDR <0.05. Based on these DEGs, we then applied the “clusterProfiler” R package to perform the functional enrichment analysis of Gene Ontology (GO), which consists of biological processes, cellular component, and molecular functions. The Kyoto Encyclopedia of Genes and Genomes (KEGG) analysis was also conducted using the same method.

### Immune, Stem Cell-Like Features and M6A Correlation Analysis

We used the single-sample gene set enrichment analysis (ssGSEA) to further assess the infiltration scores of 16 immune cells and the activity of 13 immune-related functions using the “gsva” R package (Rooney et al., 2015).

Potential immune checkpoints retrieved from previous published literatures (Tang et al., 2021) were applied to investigate the correlation between immune checkpoint–related genes and risk signatures using Wilson’s test. Tumor immunological phenotype (TIP) is an emerging concept to assess the immunological heterogeneity according to the relative infiltration of immune cells, and tumors are generally categorized into two TIPs: “hot” (inflamed) and “cold” (non-inflamed) (Nagarsheth et al., 2017). A total of 12 hot tumor–related genes and three cold tumor–related genes (Wang et al., 2021) were extracted, and correlations between the risk signature and TIP-related genes were evaluated using Wilson’s test. Spearman correlation analysis was carried out to examine the relationship between the risk score and tumor stemness.

Several studies in recent years have confirmed that 13 m6A regulator genes could influence tumor development, which comprise the “writers” (KIAA1429, METTL3, METTL14, RBM15, WTAP, and ZC3H13), the “erasers” (ALKBH5 and FTO), and the “readers” (HNRNPC, YTHDC1, YTHDC2, YTHDF1, and YTHDF2) (Wang et al., 2020a; Zhao et al., 2021). The relationship between m6A-related genes and risk signatures was evaluated using Wilson’s test.

### Drug Sensitivity Analysis

The NCI-60 database and information on 218 Food and Drug Administration–approved drugs were obtained from the Cell Miner interface (https://discover.nci.nih.gov/cellminer). NCI-60 is a free online database of nine cancer types and 60 cancer cell lines, which contains mRNA expression levels and corresponding z scores of cell sensitivity data (GI50) after drug treatment. A Pearson correlation analysis was then conducted to investigate the association between drug sensitivity and the prognostic ferroptosis-related hub genes.

### Analysis of the Protein Expression of Prognostic Ferroptosis-Related Hub Genes Between Normal Liver Tissue and Hepatocellular Carcinoma Tissue Using Immunohistochemistry

The Human Protein Atlas (HPA, version: 21.0) (Uhlén et al., 2015; Uhlen et al., 2017) is an open online database which comprises various protein expression images in normal and tumor tissues. The immunohistochemistry images of the corresponding genes in the prognosis model were obtained from the HPA database to verify the bioinformatics analysis results in our study.

### Statistical Analysis

The Student’s t test was used to identify gene expression differences between tumor and normal tissues, while the chi-square test was employed to compare differences in proportions. The OS between subgroups was compared using Kaplan–Meier analysis with the log-rank test. Univariate and multivariate Cox regression analyses were conducted to determine the independent predictors of OS. Comparisons of the ssGSEA scores of immune cells or pathways, immune checkpoints, TIP-related genes, and m6A-related genes between the high- and low-risk groups were drawn using the Wilcoxon test. Spearman correlation analysis was used to measure the relationship between the risk score and tumor stemness. Pearson correlation analysis was conducted to explore the correlation between drug sensitivity and the signature. All statistical analyses were executed using R software (Version 4.1.0). *p* value <0.05 was set as statistically significant.

## Results

In total, 377 HCC samples in the TCGA cohort and 260 HCC samples in the ICGC cohort were incorporated into the study. The detailed clinicopathological characteristics of these patients are listed in Table 1.

**TABLE 1:** Clinical characteristics of patients in the TCGA and ICGC cohorts.

Characteristic	TCGA cohort	ICGC cohort
n	377	260
Age (median, range)	61 (16–90)	69 (31–89)
Gender (%)		
Female	122 (32.4%)	68 (26.2%)
Male	255 (67.6%)	192 (73.8%)
TNM stage		
I	175 (46.4%)	40 (15.4%)
II	87 (23.1%)	117 (45.0%)
III	86 (22.8%)	80 (30.8%)
IV	5 (1.3%)	23 (8.8%)
Unknown	24 (6.4%)	0 (0.0%)
Grade		
Grade 1	55 (14.6%)	NA
Grade 2	180 (47.7%)	NA
Grade 3	124 (32.9%)	NA
Grade 4	13 (3.4%)	NA
Unknown	5 (1.3%)	NA
Status		
Alive	245 (65.0%)	214 (82.3%)
Dead	132 (35.0%)	46 (17.7%)

### Candidate Prognostic Ferroptosis-Related Hub Gene Screening in the Cancer Genome Atlas Cohort

**FIGURE 1:**
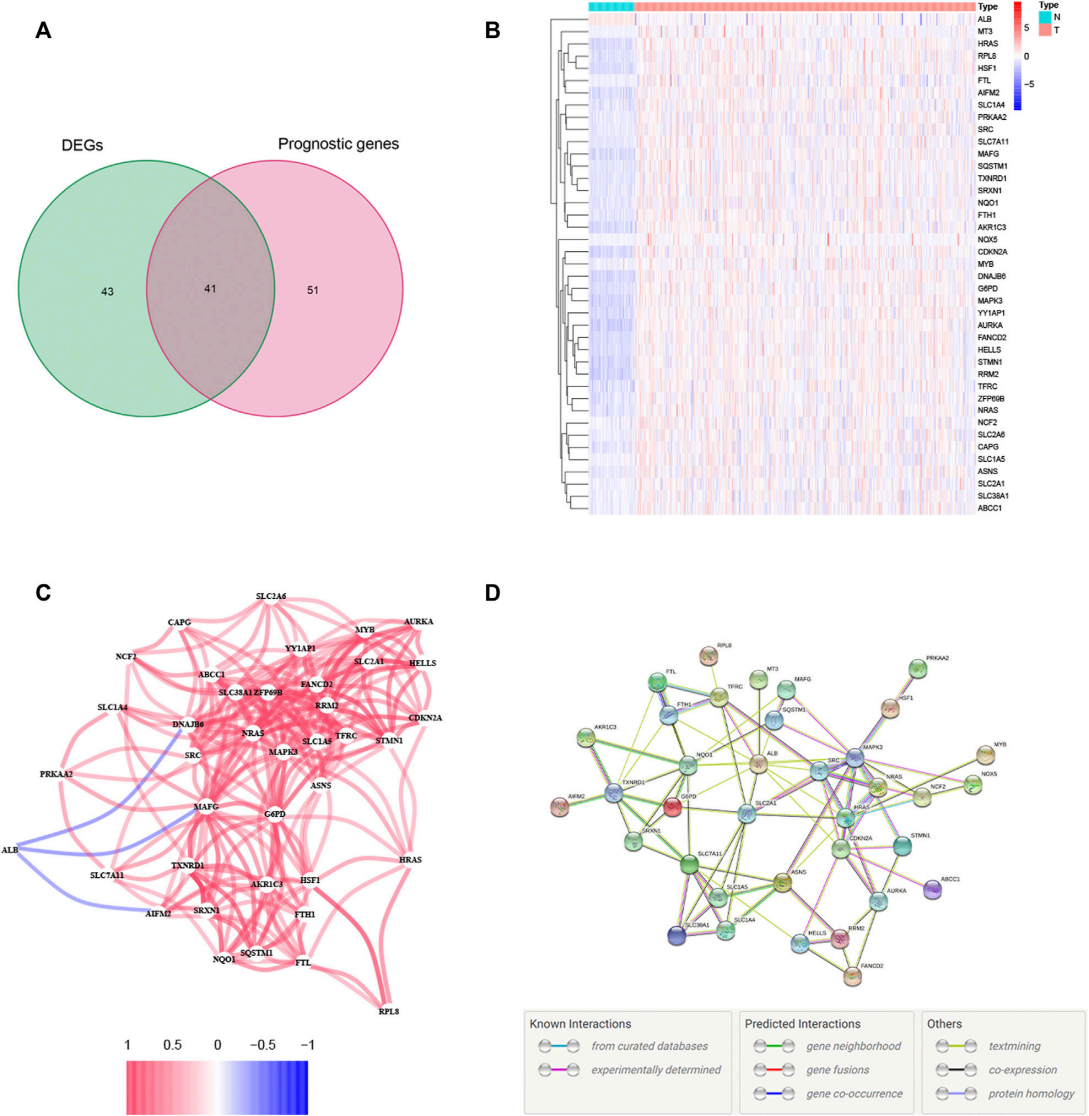
Identification of the candidate ferroptosis-related genes (FRGs) with differential expression and prognostic value in the TCGA cohort. **(A)** Venn diagram showing identified ferroptosis-related DEGs with prognostic value. **(B)** Heatmap showing the expression of the 41 overlapping genes between tumor and adjacent tissues. **(C)** The correlation network of candidate genes. The red line represents the positive correlation, while the blue line represents the negative correlation. **(D)** The PPI network indicated the interactions among the candidate genes.

**FIGURE 2:**
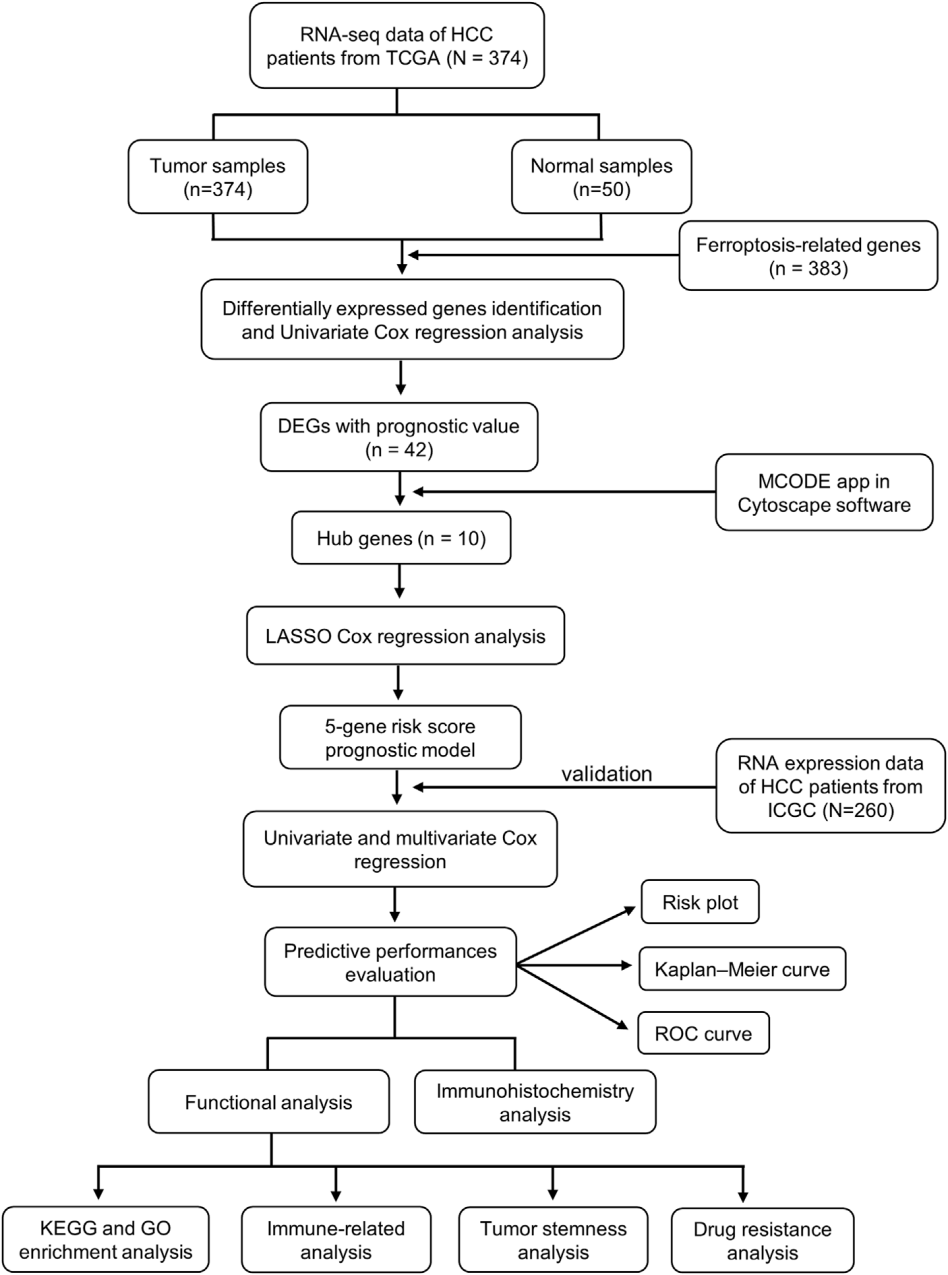
The flow diagram of data collection and analysis in the present study.

A total of 84 ferroptosis-related DEGs were identified in HCC, and 41 of them were associated with OS (Figure 1A). Among the 41 prognostic FRDEGs, all of them were upregulated in tumor tissue except ALB, which was visualized using a heatmap (Figure 1B). We used STRING and Cytoscape to find the intrinsic connections between these 41 genes. The interaction network and correlation among these genes are shown in Figures 1C,D. The hub genes including SRC, ALB, HRAS, SLC2A1, NRAS, CDKN2A, MAPK3, FANCD2, HELLS, and RRM2 were identified using Cytoscape, implying that these genes were key components of this biological network. The detailed flow diagram of this study is shown in Figure 2.

### Development of the Prognostic Model in The Cancer Genome Atlas Cohort

**FIGURE 3:**
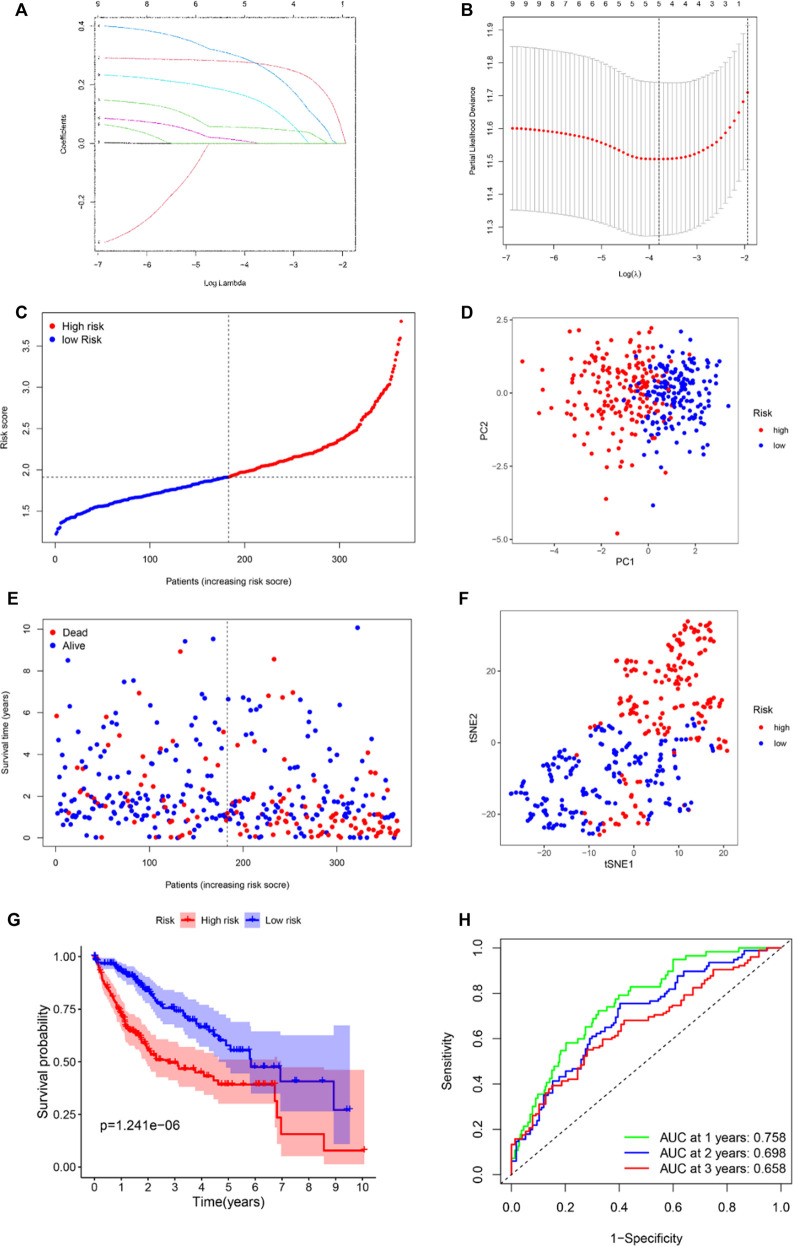
Establishment of ferroptosis-related hub gene signature in the TCGA set. **(A)** LASSO coefficient profiles of the expression of 41 candidate genes. **(B)** Selection of the penalty parameter (λ) in the LASSO model *via* 10-fold cross-validation. An optimal log λ value is indicated by the vertical black line in the plot. Risk score distribution **(C)**, PCA plot **(D)**, overall survival (OS) status **(E)**, and t-SNE **(F)** analysis of TCGA cohort. **(G)** Kaplan–Meier curves for comparison of the OS between low- and high-risk groups. **(H)** Receiver operating characteristic (ROC) curves verified the prognostic performance of the risk score.

To evaluate the prognostic value of the 10 aforementioned hub genes, we further used LASSO Cox regression analysis to set up a prognostic model. Finally, five genes, namely, HRAS, SLC2A1, NRAS, MAPK3, and RRM2, were identified based on the penalty parameter (λ) determined by the minimum criteria. The risk score was calculated using the formula: risk score = (0.151 × Exp HRAS) + (0.273 × Exp SLC2A1) + (0.276 × Exp NRAS) + (0.003 × Exp MAPK3) + (0.053 × Exp RRM2). According to the median risk score, the patients in the TCGA cohort were stratified into either high- or low-risk groups (Figure 3C). The results of the PCA and t-SNE analysis implied that the patients in the different risk groups were well distributed between two trends (Figures 3D,F). As shown in Figure 3E, patients with high risk had a higher rate of earlier death and poorer survival time than those with low risk. The survival analysis between the two groups is displayed in Figure 3G. Notably, the OS in the high-risk group was lower (*p* < 0.001). The time-dependent receiver operating characteristic (ROC) curve was used to evaluate the predictive ability of the model, and the area under the curve (AUC) reached 0.758 at 1 year, 0.698 at 2 years, and 0.658 at 3 years (Figure 3H).

### External Validation of the Risk Signature in the International Cancer Genome Consortium Cohort

**FIGURE 4:**
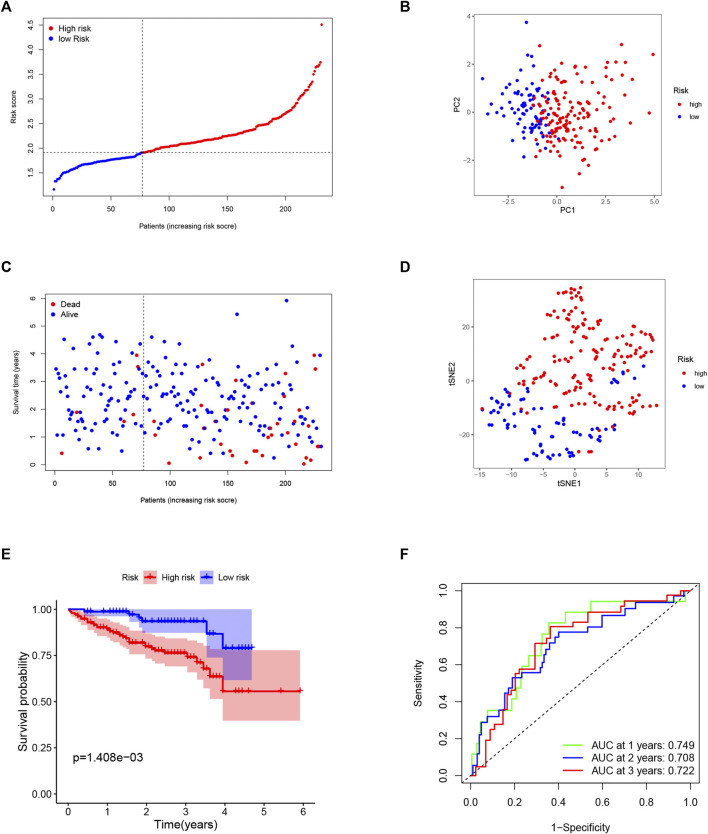
Validation of the 5-gene signature in the ICGC cohort. **(A)** The distribution and median value of the risk scores in the ICGC cohort. **(B)** The distributions of overall survival (OS) status. **(C)** PCA plot of the ICGC cohort. **(D)** t-SNE analysis of the ICGC cohort. **(E)** Kaplan–Meier curves for the OS of patients in the high- and low-risk groups. **(F)**. ROC curves in the ICGC cohort.

To test the reliability of the model established using the TCGA cohort, the patients from the ICGC cohort were also categorized into low- or high-risk groups by the median risk score calculated with the same formula used for the TCGA cohort. Similarly, PCA and t-SNE analysis also showed excellent separations between the two groups (Figures 4B,D). We also found that patients in the high-risk group had a higher possibility of encountering earlier death (Figure 4C) and had a significantly lower survival possibility than those in the low-risk group (Figure 4E). Meanwhile, the time–ROC curve also showed great predictive ability of our model in the ICGC cohort, and the AUC predictive value of the 5-gene signature for 1-, 2-, and 3-year survival rates was 0.749, 0.708, and 0.722, respectively (Figure 4F).

### Independent Prognostic Value of the Risk Model

**FIGURE 5:**
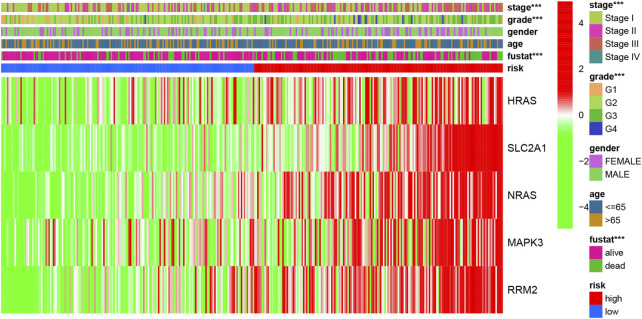
The heatmap of clinicopathological features and ferroptosis-related hub gene expression in two risk subgroups.

**FIGURE 6:**
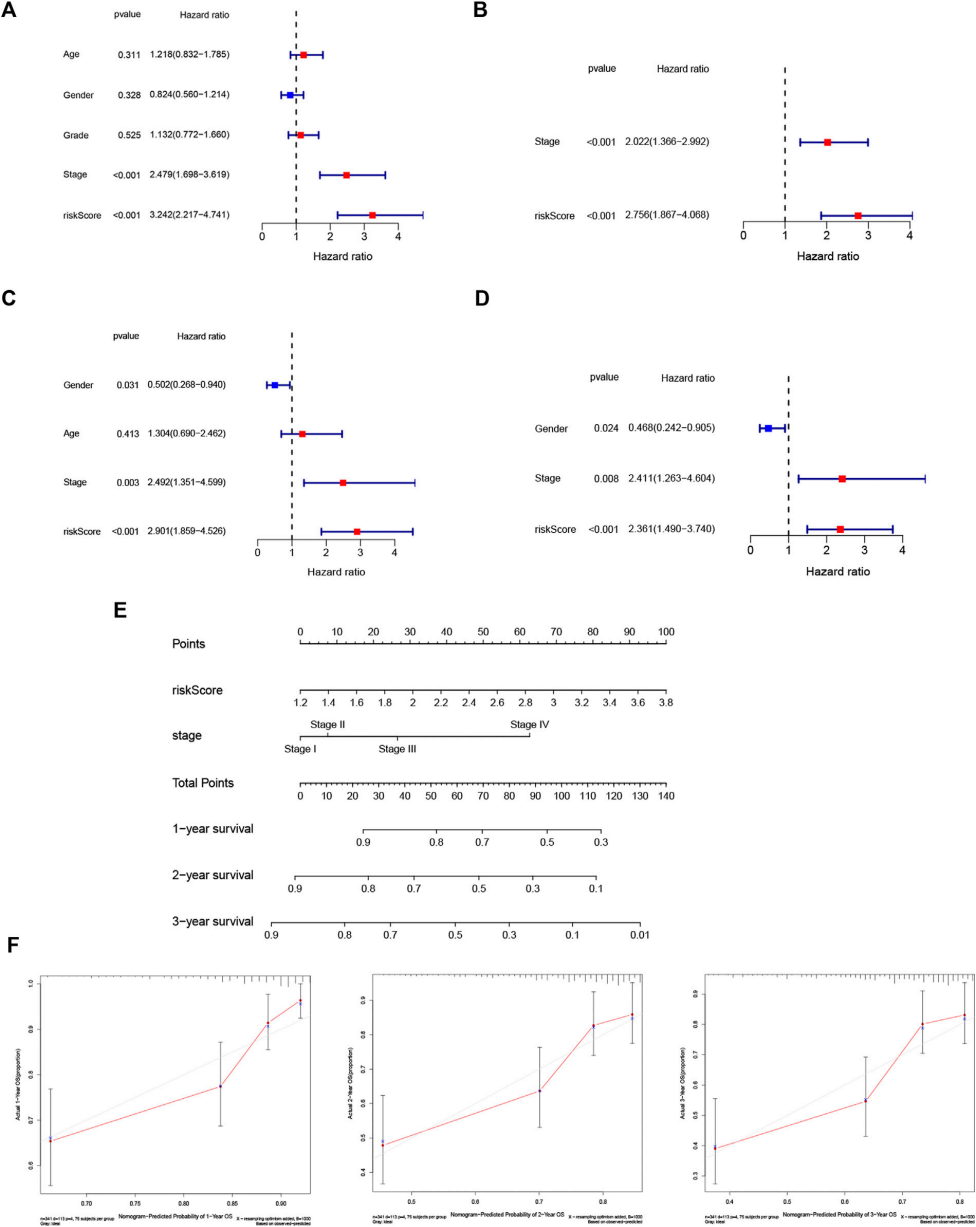
Risk score is an independent prognostic signature for HCC and nomogram with calibration curves of the prognostic factor screened by multivariate Cox regression. Results of the univariate **(A)** and multivariate **(B)** Cox regression analyses of OS in the TCGA cohort. Univariate **(C)** and multivariate **(D)** Cox regression analyses of OS in the ICGC cohort. **(E)** Nomogram of the TCGA cohort. **(F)** Calibration curve of the TCGA cohort.

A heatmap of clinical characteristics and risk subgroups in the TCGA cohort is shown in Figure 5. All five genes (HRAS, SLC2A1, NRAS, MAPK3, and RRM2) were upregulated in the low-risk subgroup. Univariate and multivariable Cox regression analyses were generated to determine whether a signature-based risk score could be an independent prognostic indicator. The risk score was significantly associated with OS in the TCGA (HR = 3.242, 95% CI: 2.217–4.741, Figure 6A) and ICGC cohort (HR: 2.901, 95% CI: 1.859–4.526, Figure 6C) according to univariate regression analysis results. Subsequently, multivariate Cox regression analysis demonstrated that the risk scores were independent predictors connected with OS (TCGA cohort: HR = 2.756, 95% CI = 1.867–4.068, *p* < 0.001; ICGC cohort: HR = 2.361, 95% CI = 1.490–3.740, *p* < 0.001; Figures 6B,D).

### Establishment of the Nomogram

To better apply the signature to clinical practice, we developed a nomogram based on risk score and other independent prognostic factors (TNM stage) in the TCGA cohort (Figure 6E). Moreover, calibration plots of 1-, 2- and 3-year survival probabilities also showed excellent consistency between nomogram predictions and actual observations (Figure 6F).

### Functional Analyses

In order to figure out the biological functions and pathways related to the risk score, we quantified the enrichment analysis of GO and KEGG pathways in high-risk and low-risk patients in the TCGA and ICGC cohorts.

**FIGURE 7:**
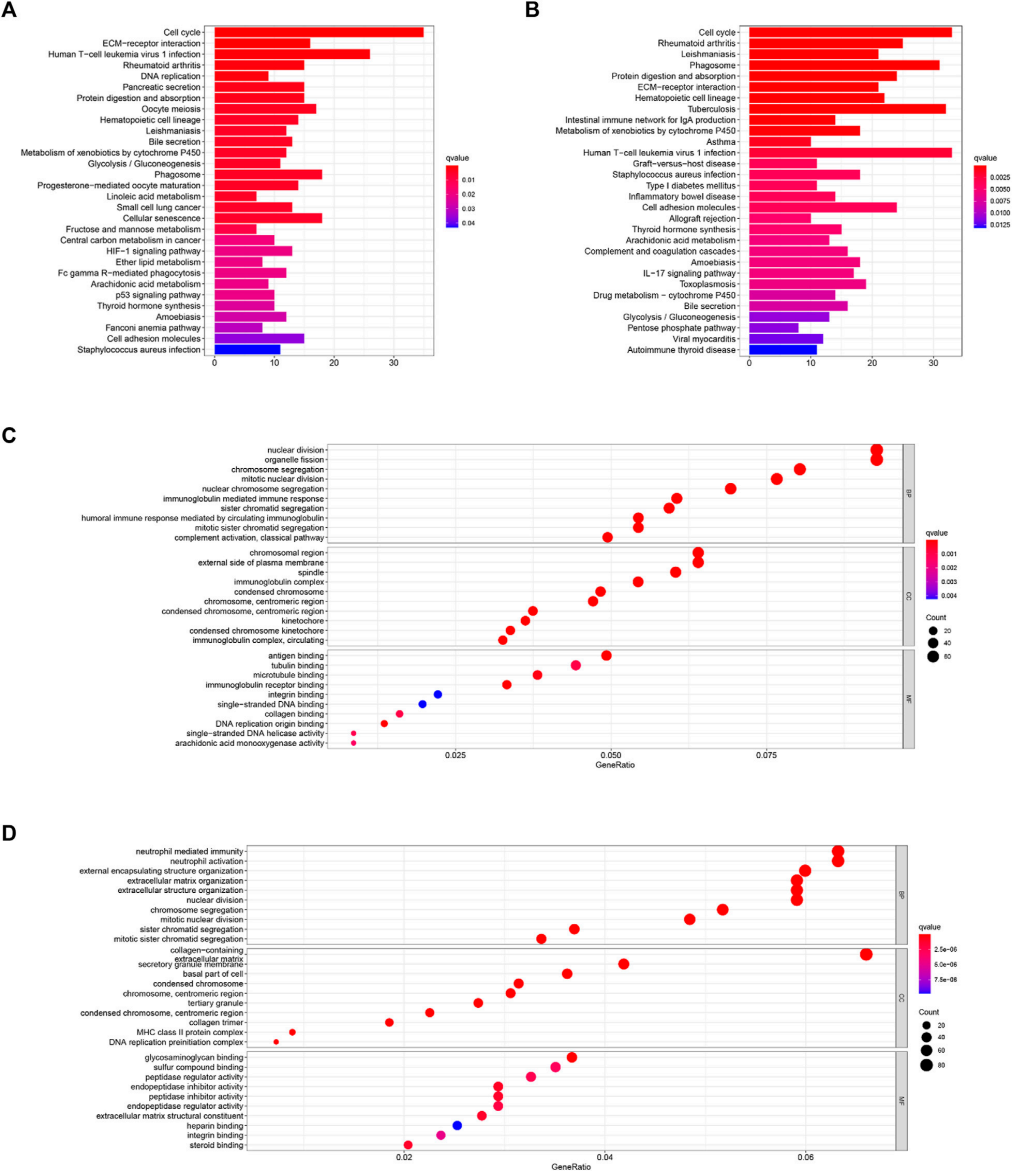
KEGG **(A,B)** and GO **(C,D)** enrichment analyses were conducted between the high- and low-risk groups in the TCGA **(A,C)** and ICGC **(B,D)** cohorts.

As shown in Figures 7C,D, GO enrichment analysis between the two cohorts was significantly enriched in nuclear division and mitotic nuclear division. KEGG pathway analysis (Figures 7A,B) revealed that these DEGs were closely associated with the cell cycle, human T-cell leukemia virus infection, the metabolism of xenobiotics by cytochrome P450, extracellular matrix (ECM)–receptor interactions, and so on. Thus, these results indicate the correlation between ferroptosis and these essential biological processes.

### Associations With Immunity, Tumor Stemness, and M6A-Related Genes

**FIGURE 8:**
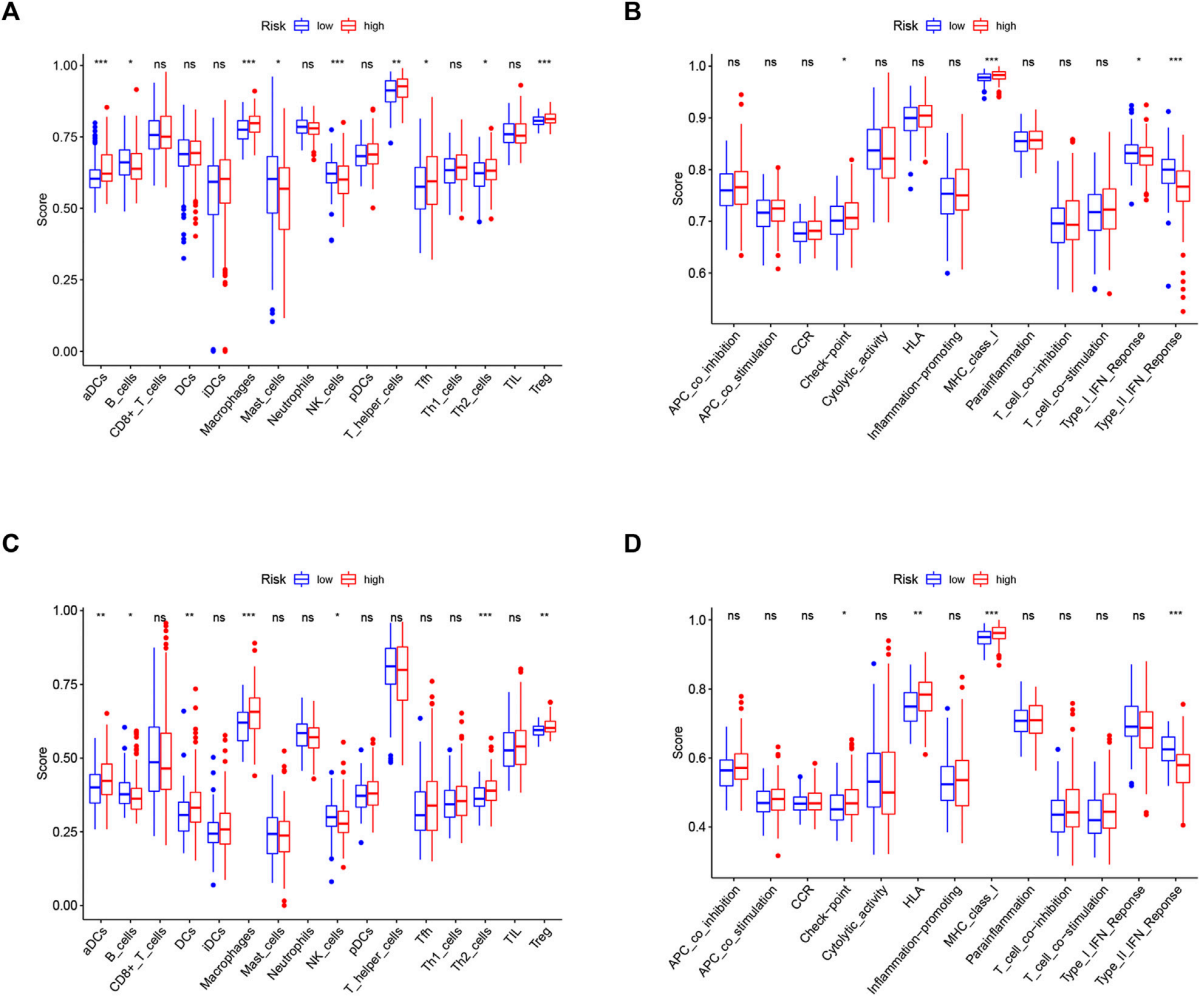
Comparison of the ssGSEA scores between different risk groups in the TCGA cohort **(A,B)** and ICGC cohort **(C,D)**. The scores of 16 immune cells **(A,C)** and 13 immune-related functions **(B,D)** are displayed in boxplots. CCR, cytokine–cytokine receptor. Adjusted *p* values were shown as ns, not significant; *, *p* < 0.05; **, *p* < 0.01; and ***, *p* < 0.001.

Since ferroptosis is linked with tumor immunity and can affect the outcomes of tumor immunotherapy, it is worth calculating the enrichment scores of diverse immune cells, related functions, or pathways using ssGSEA in both the TCGA and ICGC cohorts. As shown in Figures 8A,C, aDCs, macrophages, T helper cells, Th2 cells, and Treg cells showed high infiltration in the high-risk group in both TCGA and ICGC cohorts (all *p* < 0.05). With respect to the immune-related pathways, checkpoint and MHC class I were significantly upregulated in the high-risk group, while type I IFN response and type II IFN response were opposite in the TCGA cohorts (all adjusted *p* < 0.05, Figure 8B). In the ICGC cohorts, checkpoint, HLA, and MHC class I were significantly upregulated in the high-risk group, while type II IFN responses were converse (all adjusted *p* < 0.05, Figure 8D). These enriched immune-related pathways implied that the ferroptosis participates in the development of tumor immune evasion.

**FIGURE 9:**
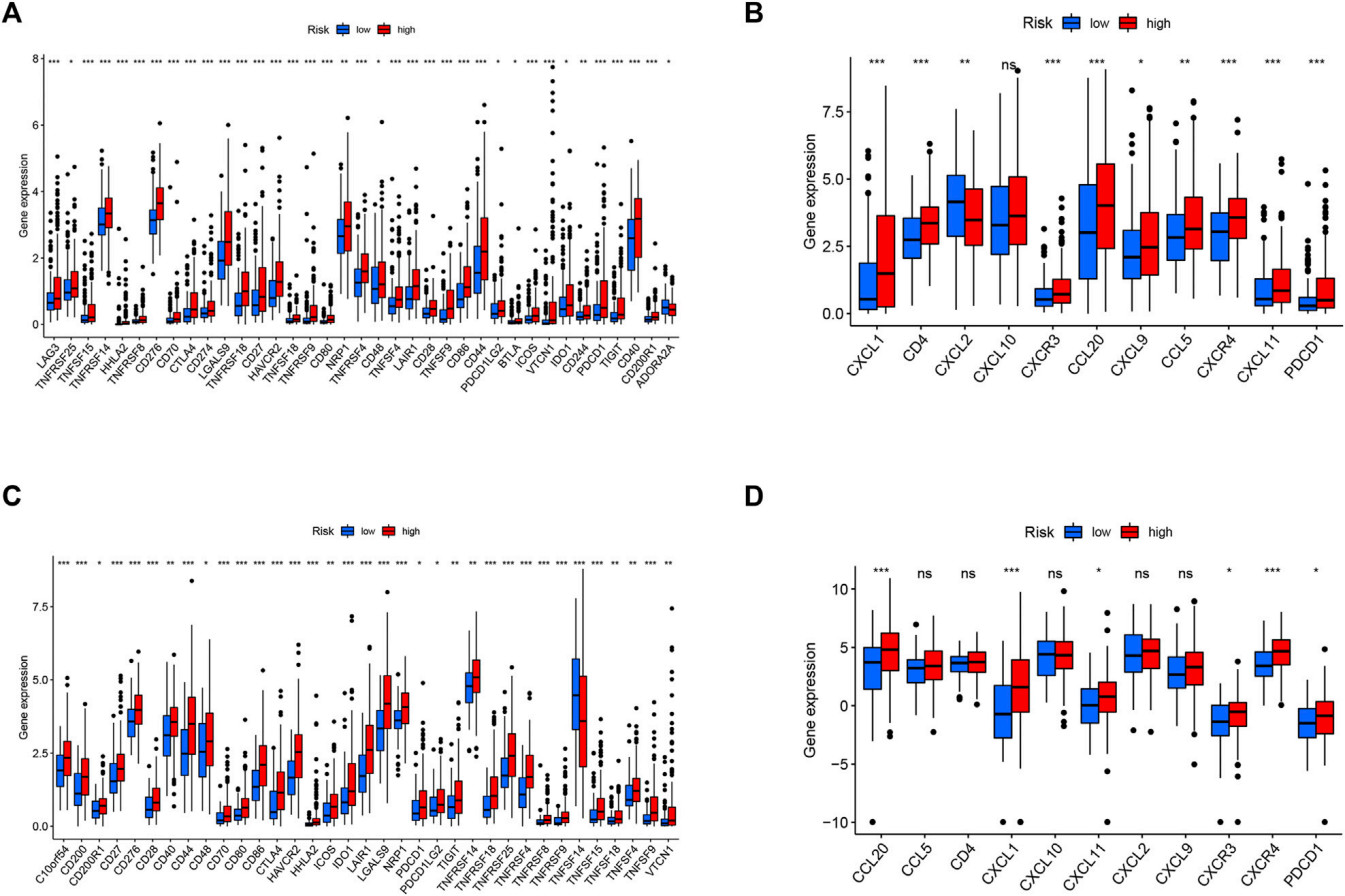
Correlation between the risk score, immune checkpoints, and TIP-related genes. Expression of immune checkpoints between two risk subgroups in the TCGA cohort **(A)** and ICGC cohort **(C)**. The expression of TIP-related genes between high- and low-risk groups in the TCGA cohort **(B)** and ICGC cohort **(D)**. Adjusted *p* values were shown as *, *p* < 0.05; **, *p* < 0.01; and ***, *p* < 0.001.

To the best of our knowledge, tumor cells can escape from immune surveillance and promote tumor growth and progression through the activation of distinct immune checkpoint pathways. Considering the important role of immune checkpoints in immunotherapy, we further explored the difference in immune checkpoint expression between the two groups. The expression levels of all identified immune-related genes were higher in the high-risk subgroup, except for ADORA2A in the TCGA cohort and TNFSF14 in the ICGC cohort. We also found an obvious difference in the expression of PDCD1 (PD-1), CTLA-4, and HHLA2 between the two groups of patients (Figures 9A,C), indicating a potential role of the risk model in predicting immune responses to immunotherapy in HCC patients.

Tumor immunological phenotype has been reported to be significantly associated with prognosis and therapeutic responses in various cancers by accumulating evidence (Fu et al., 2018; Zhou et al., 2019a; Wang et al., 2020b; Sui et al., 2020; Wang et al., 2021). Wang and coauthors recently reported 12 hot tumor-related genes (CXCR3, CXCR4, CXCL9, CXCL10, CXCL11, CCL5, CD3, CD4, CD8a, CD8b, CD274, and PDCD1) and three cold tumor-related genes (CXCL1, CXCL2, and CCL20) constitute the TIP gene signature using a text-mining approach (Wang et al., 2021), which is significantly associated with the survival outcomes of cancer patients and presents better predictive ability in immunotherapeutic responses than widely used immune signatures such as tumor mutation burden (TMB) and tumor immune dysfunction and exclusion (TIDE). Hence, we analyzed the relationships between the signature and TIP-related genes, and the results showed that cold tumor genes such as CXCL2 and CCL20 and hot tumor genes such as CXCR3, CXCL11, and PDCD1 were upregulated in the high-risk group in both cohorts (Figures 9B,D).

**FIGURE 10:**
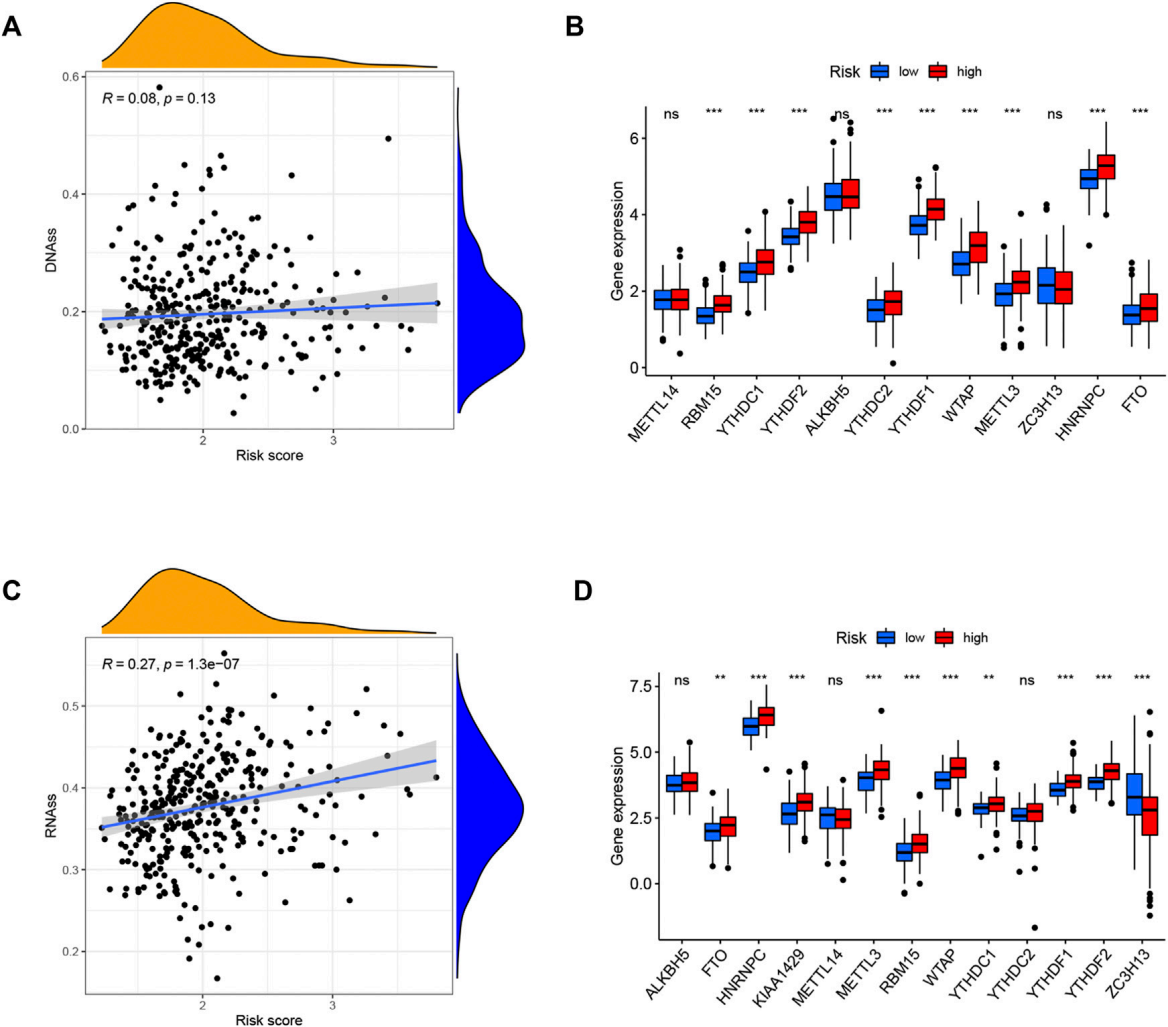
Potential role of risk signature in HCC tumor stemness and m6A-related genes. Associations between risk signature and DNAss **(A)**, RNAss **(C)**. Comparison of the m6A-related genes between different risk groups in the TCGA cohort **(B)** and ICGC cohort **(D)**.

Tumor stemness (including the RNA stemness score and DNA methylation pattern) and m6A-related genes are critical regulators of tumor progression. The established risk signature was significantly positively correlated with RNA methylation patterns (RNAss; Figures 10A,C). In addition, the expression levels of m6A-related genes FTO, HNRNPC, METTL3, RBM15, WTAP, YTHDC1, YTHDF1, and YTHDF2 were significantly higher in the high-risk subgroup than in the low-risk subgroup in both TCGA and ICGC cohorts (Figures 10B,D). These findings imply that these ferroptosis-related hub genes may be closely associated with the immune state of HCC.

### Relationship Between Prognostic Ferroptosis-Related Hub Genes and Drug Sensitivity

**FIGURE 11:**
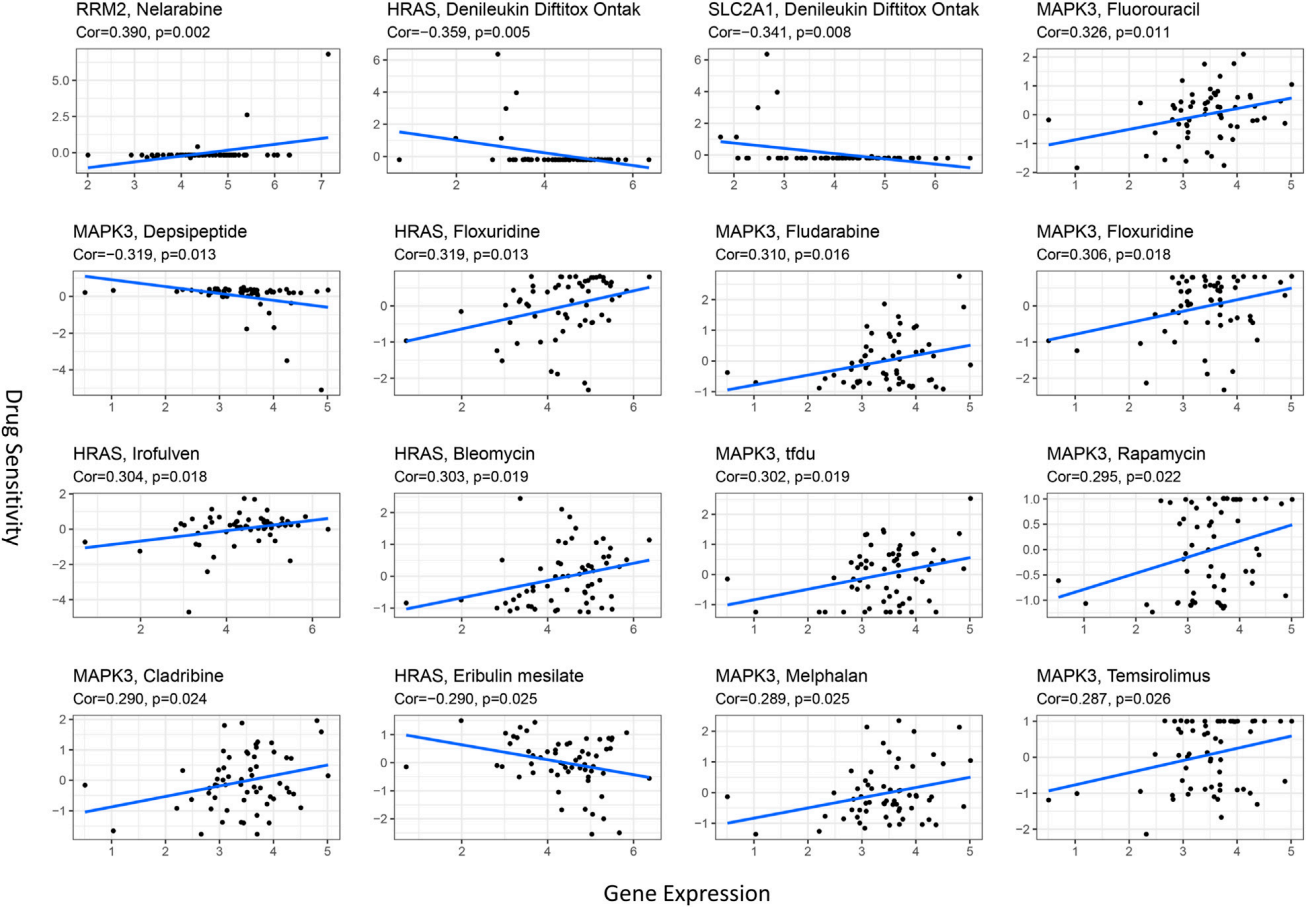
Scatter plots for the association between prognostic hub gene expression in the model and drug sensitivity (top 16 ranked by *p*-value).

Ferroptosis has been reported to play a crucial role in modulating drug resistance. Herein, we used the NCI-60 database to explore the connection between prognostic ferroptosis-related hub genes and drug sensitivity using Pearson correlation. The top 16 gene–drug pairs ranked by Pearson correlation coefficient are displayed in Figure 11. We found that RRM2 was positively associated with chemotherapy sensitivity, while SLC2A1 was negatively associated with targeted drug sensitivity. Intriguingly, MAPK3 was positively related to eight drug sensitivity tests, including chemotherapy and targeted drugs. These results proved that ferroptosis was involved in targeted therapies in HCC.

### Differences in the Protein Expression of Five Prognostic Ferroptosis-Related Hub Genes Between Normal Liver Tissue and Hepatocellular Carcinoma Tissue

**FIGURE 12:**
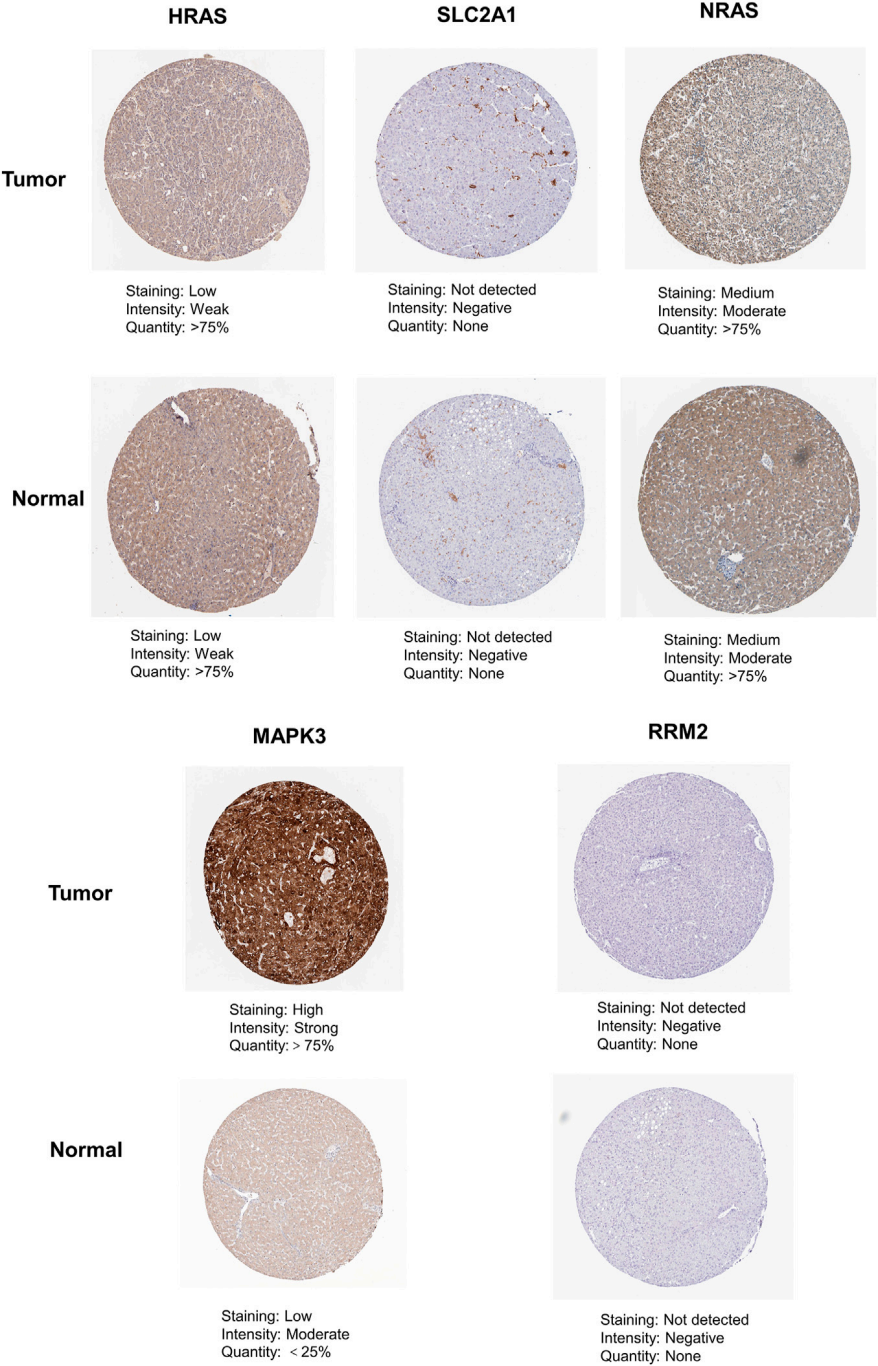
The immunohistochemistry images of related hub genes from the HPA database in liver cancer tumor tissue and normal tissues.

As shown in Figure 12, we found that the expression of MAPK3 in liver cancer tissue is higher than that in normal tissue by immunohistochemical staining in the HPA database. In contrast, the protein expression of HRAS, SLC2A1, NRAS, and RRM2 showed no significant difference between normal liver tissues and HCC tissues.

## Discussion

Despite tremendous and rapid progress in the present diagnosis and treatment of HCC, patients with HCC still have low OS rate and poor prognosis (Anwanwan et al., 2020). Hence, it is necessary to identify new biomarkers and targets that affect the prognosis of HCC, so as to optimize the early diagnosis of HCC and improve treatment to enhance the clinical efficacy against HCC. Distinct from apoptosis, autophagy and necrosis, ferroptosis is a novel form of programmed cell death characterized by unique morphology, gene expression, and molecular pathways (Tang et al., 2019). Though the underlying mechanisms of tumor susceptibility to ferroptosis have been a research hotspot over the past few years, the potential regulatory roles between ferroptosis and tumor immunity have not been systemically studied.

Previously, FRG signatures have been established and applied to HCC in recent research (Liang et al., 2020; Wan et al., 2022). In contrast to previous studies, we screened out the ferroptosis-related hub genes and made the model we built more targeted. Moreover, we performed a comprehensive analysis of the relationship between ferroptosis-related hub genes and liver cancer. We not only constructed the prognostic model, but also emphasized the analysis between ferroptosis and immunotherapy in HCC.

In the present study, we constructed a ferroptosis-related model consisting of five Hub genes (HRAS, SLC2A1, NRAS, MAPK3, and RRM2) for predicting the prognosis of HCC according to the data from TCGA and verified its predictive ability in the ICGC cohort. Among the five genes in the risk signature, HRAS is a small G protein in the RAS subfamily of the RAS superfamily of small GTPases. The expression level of HRAS was higher in HCC cell lines and HCC tissues, suggesting a transcriptional activation mechanism of HRAS in HCC rather than oncogenic mutations (Dietrich et al., 2017). Moreover, activated HRAS mutations were detected in nonalcoholic fatty liver disease (NAFLD)–associated HCC in mice (Shen et al., 2016), which is increasingly regarded as a promotor of hepatocarcinogenesis.

Solute carrier family 2 member 1 (SLC2A1), also known as glucose transporter 1 (GLUT1), is an energy source for cell growth that lends favor to cancer development and progression (Min et al., 2021). In addition, SLC2A1-mediated glucose transport facilitates glycolysis, accelerates fatty acid synthesis, and eventually induces lipid peroxidation–dependent ferroptosis (Song et al., 2021). High expression of SLC2A1 has been found to be connected with inferior outcomes in various adult malignancies, including liver cancer (Chen et al., 2018), lung cancer (Zhang et al., 2019), breast cancer (Deng et al., 2018), colorectal cancer (Yang et al., 2017), and so on. Furthermore, it has been revealed that all-trans-retinoic acid (ATRA) could be a candidate drug for the treatment of gastric cancer patients with high SLC2A1 expression and resistance to conventional chemotherapy (Min et al., 2021).

A previous transcriptome profiling study demonstrated that neuroblastoma RAS viral oncogene homolog (NRAS) was dysregulated in fibrolamellar HCC, although the functions and clinical implications of NRAS were unknown (Sorenson et al., 2017). Another recent study found that NRAS and c-MYC are simultaneously upregulated by insulin-like growth factor II in HCC, but the specific function of NRAS was not investigated (Ji et al., 2017). Additionally, NRAS overexpression was related to poor survival and proliferation *in vivo*. NRAS knockdown increased the efficacy of sorafenib in resistant cells and may be a promising prognostic predictor in HCC (Dietrich et al., 2019).

MAPK3, also called extracellular signal-regulated kinase-1 (ERK-1), is a protein that plays a critical part in the ERK signaling pathway. Specifically, it regulates cell proliferation, cycle, and apoptosis (McCubrey et al., 2007). Previous studies revealed that MAPK3 expression was upregulated in human HCC cells (Schmidt et al., 1997) and was related to drug resistance (Yan et al., 2009; Zhang et al., 2009). Similarly, Bendix and coauthors found that MAPK3 enabled the regulation of the activation of natural T cells by dendritic cells (DC) (Bendix et al., 2010).

Ribonucleotide reductase M2 subunit (RRM2) is a rate-limiting enzyme related to DNA synthesis and damage repair, which plays a momentous role in many crucial cellular processes including cell proliferation, invasiveness, migration, and angiogenesis (Nordlund and Reichard, 2006). It has been observed that the expression of RRM2 in HCC tissues was higher than that in normal tissues, and an anti-RRM2 siRNA duplex could inhibit proliferative activity in HCC (Gao et al., 2013). Moreover, Zhou and coauthors revealed that RRM2 overexpression was closely related to poor prognosis of HCC patients, and RRM2 was enriched in the p53 signaling pathway (Zhou et al., 2019b). RRM2 has been reported to be an independent predictor of early recurrence of HCC, indicating that RRM2 may facilitate tumor cells metastasis (Lee et al., 2014). According to recent reports, RRM2 could antagonize ferroptosis in liver cancer cells by sustaining glutathione (GSH) synthesis, which is a promising biomarker for the diagnosis of liver cancer (Yang et al., 2020a). Sorafenib is the first FDA-approved systemic molecular targeted therapy drug for advanced HCC, which can induce ferroptosis of cancer cells in HCC (Louandre et al., 2013). Interestingly, Yang and coworkers demonstrated that RRM2 overexpression partially rescues HCC cells from the cytotoxicity of sorafenib, and RRM2 is a novel target of sorafenib in HCC (Yang et al., 2020b).

We further employed functional analysis and found that HCC-related biological processes such as nuclear division and the cell cycle were enriched. In addition, we found that the signature was significantly associated with immune cell infiltration and enriched in immunity-related pathways in HCC patients. In addition, we also found a substantial difference in TIP-related genes between high-risk and low-risk groups. Currently, cancer therapy has entered the era of immunity and iron (Tarangelo and Dixon, 2016; Jiang et al., 2019). Nanoparticles regulate iron and reactive oxygen species (ROS) levels to induce ferroptosis, providing a promising therapeutic strategy for cancer therapy (Xu et al., 2019). Immunotherapy has become a new criterion for treatment for advanced HCC worldwide (Wang and Wang, 2019). However, only a small proportion of HCC patients can respond to immunotherapies (Ramos-Casals et al., 2020), and the selection of available and suitable targets for individualized therapy remains a difficult problem for HCC patients. In our study, we also evaluated the correlation of the signature with response to immunotherapy. We discovered that PD-1 and CTLA-4 were dramatically upregulated in the high-risk group, indicating that immune checkpoint inhibitors could be more effective in HCC patients with the high-risk signature score. Although there are no available drugs or clinical trials targeting the identified hub genes at present, our findings suggest that ferroptosis may open a new chapter in the immunotherapy of tumors.

Cancer stem cell–like cells (CSCs) promote tumor growth due to their self-renewal and invasive abilities. In the current study, the risk signature was positively correlated with the stem cell score, confirming that our newly identified gene signature was a risk factor for HCC. M6A-related genes have also been an active area of recent tumor research (Liao et al., 2021). Our signature could accurately predict the expression levels of the m6A-related genes FTO, HNRNPC, METTL3, RBM15, WTAP, YTHDC1, YTHDF1, and YTHDF2 in HCC. However, the potential mechanisms of these relationships require further investigation.

Resistance of cancer cells to chemotherapy is a paramount challenge in cancer treatment. Ferroptosis inducers may provide new avenues to the problem of tumor drug resistance as they could overcome the disadvantages of traditional chemotherapeutic agents (Shen et al., 2018). Hence, we conducted drug sensitivity analysis and demonstrated that ferroptosis had a close correlation with chemotherapy resistance. Intriguingly, the results also provided a novel perspective that ferroptosis may be involved in some tumor-targeted therapy resistance.

However, many key issues such as the interrelation between ferroptosis and other cell deaths and host immunogenicity remain poorly understood. Therefore, our analysis offered new insights into the creation of effective clinical diagnostic and therapeutic strategies in HCC as well as a theoretical basis for future research. It is much less clear about the potential mechanisms between ferroptosis-related hub genes and tumor immunity in HCC and warrants further exploration.

Nevertheless, a few limitations of this study should be taken into consideration. First, we utilized retrospective data from public databases to construct and validate our prognostic model. Thus, some bias is unavoidable, and different cohorts are needed to validate its clinical utility henceforth. Second, the current study only included simple database analysis without any validation through experimental research to prove our conclusions. Therefore, the reliability of our results cannot be fully guaranteed.

## Conclusion

In summary, our study established and verified a hub gene signature associated with ferroptosis that can precisely predict the prognosis of HCC patients. In addition, our proposed signature was closely associated with tumor immunity and drug resistance. Our study can not only provide innovative biomarkers for accessing HCC prognosis and uncover important evidence for future research on the mechanisms between ferroptosis-related hub genes and the immunity of liver cancer, but also offer new insights into drug resistance in liver cancer and can significantly guide improvements in the treatment of liver cancer.

## Data Availability

The original contributions presented in the study are included in the article/Supplementary Material; further inquiries can be directed to the corresponding author.
